# Indian herb *Tinospora cordifolia* and *Tinospora* species: Phytochemical and therapeutic application

**DOI:** 10.1016/j.heliyon.2024.e31229

**Published:** 2024-05-16

**Authors:** Anu Chaudhary, Rina Das, Kiran Mehta, Dinesh Kumar Mehta

**Affiliations:** a Department of Pharmaceutical Chemistry, MM College of Pharmacy, Maharishi Markandeshwar (Deemed to be University), Mullana, Ambala, 133207, India; b Chitkara Business School, Chitkara University, Rajpura, 140401, India

**Keywords:** *Tinospora cordifolia*, *Tinospora* species (*Menispermaceae*), Phytochemistry, Pharmacological, Safety, Toxicity

## Abstract

Clinical investigations are increasingly focusing on natural materials with medical benefits because, in contrast to medicines, they have extremely few adverse effects. *Tinospora* species of the *Menispermaceae* family has many bioactive principles for plant nutraceuticals. A thorough assessment of the existing literature revealed that Indian *Tinospora* species are an important group of medicinal herbs used for a variety of pharmacological activities. While, *Tinospora cordifolia* is widely recognized as a significant herb in the Indian System of Medicines (ISM) due to its bioactive components and has been used in the treatment of diabetes, cancer, urinary problems, fever, jaundice, helminthiasis, leprosy, dysentery, skin diseases, and many more.

Using the search phrases “phytochemistry,” “traditional uses,” and “pharmacological evaluation of Indian *Tinospora* species,” appropriate articles were carefully extracted from the MEDLINE/PubMed, Scopus, and WOS databases. Around 180 articles, related to the India *Tinospora* species, were selected from a pool of 200 papers published between 1991 and 2023. *T. cordifolia* has received a lot of scientific attention because of its diverse therapeutic characteristics in treating various diseases. Our present study in this review encompasses 1.) Phytochemistry, traditional uses and pharmacological potential of *T. cordifolia* as well as other Indian *Tinospora* species. 2.) Safety and toxicity study and available marketed formulation of *T. cordifolia* for the treatment of various diseases.

The chemical constitution and pharmacological characteristics of other *Tinospora* species must also be investigated, indicating a need for further scientific research.

## Highlights

• This paper provides a comprehensive analysis of Indian *Tinospora cordifolia* and several *Tinospora* species, highlighting their diverse bioactive components and their demonstrated potential in ethnopharmacology.
• Phytochemistry and Pharmacological potential of *Tinospora* species.
• Safety profile, Toxicity study and available Marketed formulation of *T. cordifolia* for the treatment of various diseases.

## 1 Introduction

Herbal formulations are pharmaceutical preparations of one or more plants present in precise quantities to offer cosmetic, diagnostic, and therapeutic benefits to humans or animals [1].

Phytomedicine is also known as plant medicine. Because herbal medicine has fewer adverse effects and is more compatible with the human body, 70–80 percent of people continue to use it for primary health care [2,3]. India's biodiversity and extensive comprehension of traditional herbal medicine systems such as Siddha, Ayurveda, and Unani provide a solid foundation for the general healthcare application of a wide variety of species and common ailments [4].

The *Tinospora* genus encompasses approximately 34 species that are distributed over the tropical and subtropical regions of Asia, Australia, and Africa [5]. There is a total of nine *Tinospora* species that have become naturalized in various states across India. Contemporary pharmacological investigations and clinical applications have revealed that *Tinospora* species exhibit a diverse range of actions. *Tinospora cordifolia* is widely recognized as a significant herb in the Indian System of Medicines (ISM) due to its bioactive components and numerous therapeutic properties, in comparison to other Indian species such as *T. crispa* (*L*.), *T. sinensis (Lour.), T. baenzigeri.* Whereas no ethnopharmacological activity has been reported for *T. smilacina, T. maqsoodiana, T. formanii, T. glabra,* and *T. subcordata* in ISM.

*T. cordifolia* (Wild) Hook. F & Thomson, Tinospora Gulancha Indian *Tinospora*, and Giloya are its Latin names *T. cordifolia* is also known as *Tinospora sinensis* (Lour.) Merr. and Guduchi/Amrita. It is a member of the *Menispermaceae* family and is native to China, Myanmar, and Sri Lanka [6]. The herb is commonly employed in conventional ayurvedic treatment. It can be used to treat jaundice, rheumatism, urinary diseases, skin conditions, diabetes, anemia, inflammation, allergic condition, anti-periodic qualities, radioprotective properties, and other conditions [7,8]. The root of the giloya plant (*T. cordifolia*) is used as an emetic and to relieve intestinal obstruction. This plant's starch is an effective at home treatment for chronic fever, relieving stinging sensations while increasing energy and appetite [9,10].

Giloya is useful in the treatment of helminthiasis, cardiovascular illness, leprosy, rheumatoid arthritis, and other disorders [11], and it also helps keep the immune system and the body's resistance to infections strong. It's also useful for treating gastrointestinal problems like hyperacidity, colitis, worm infestations, anorexia, stomach pain, excessive thirst, vomiting, and liver illnesses like hepatitis [12,13]. The root, stem, and entire plant all contain chemical components responsible for the plant's pharmacological activities, such as glycosides, diterpenoid lactones, sesquiterpenoids, steroids, aliphatic compounds, phenolics, essential oils, polysaccharides, a mixture of fatty acids [14].

It has garnered a great deal of scientific interest due to its diverse therapeutic properties in treating various diseases. In this review, our current work looks at 1.) Phytochemistry and pharmacological potential of *T. cordifolia*. 2.) Safety profile, toxicology study, and commercially available formulations for treating different illnesses.

## 2 Materials and methods

Using the search phrases “phytochemistry,” “chemical constituents,” “traditional uses,” and “pharmacological evaluation of Indian *Tinospora cordifolia* and *Tinospora* species,” appropriate articles were carefully extracted from the MEDLINE/PubMed, Scopus, and WOS databases. Around 170–175 articles, related to the India *Tinospora* species, were selected from a pool of 200 papers published between 1991 and 2023.

### 2.1 Growth Prerequisite

Several plant species can be used for medical purposes [15]. *T. cordifolia* is quickly disappearing from its natural habitat despite its widespread use in traditional and modern medicine. Biotechnological strategies for rapid distribution, scalability of secondary metabolites, and preservation of unusual, endangered, and scarce medicinal plants should be adopted [16] because the conventional approach falls short of easing depletion. Micropropagation in vitro is one of the best alternative strategies for rapid clonal mass replication of a disease-free, high-yielding plant. In vitro, metabolite creation and a few other technological methods for producing new species require cell culture [17]. Tissue culture allows for the rapid multiplication of this plant, which is mostly produced for its aesthetic value. Its optimal conditions for growth vary little from one soil type to the next or from one set of environmental conditions to another. If the plant growing on the neem tree is trained well, it will have a higher number of medicinal properties [18]. The plant is widespread across the tropics and subtropics, while it thrives best in dry, hot areas with plenty of moisture [19]. Discovery, reproduction, and survival of these fragile genotypes may rely heavily on biotechnological instruments.

## 3 Ethnomedicinal usage of *Tinospora* species

*T. cordifolia* is utilised to protect against a variety of human health issues. Particular attention has been paid to its efficacy in treating endocrine and metabolic diseases, as well as its capacity to boost immunity [20]. There have been reports of *T. cordifolia* as a key component of medications for treating metabolic, endocrine, and several other diseases receiving current protection.

In addition to reducing vascular endothelial growth factor, anti-inflammatory and analgesic properties prevent drug-resistant HIV and HIV from binding to TNF. The plant's roots, stem (branches), leaves, starch, flowers, and fruits, among other parts, all aid the human body in the treatment of a variety of maladies. *T. cordifolia* starch is an effective treatment for soothing searing sensations, persistent fever, enhancing appetite, and enhancing vitality. The roots of this plant is used as an emetic and to treat intestinal obstruction.

This herb can be used to treat a variety of ailments, such as stomach upset, diabetes, indigestion, high cholesterol, fever, gout, cancer, including lymphoma, liver disease, rheumatoid arthritis, stomach ulcer and gonorrhea, and syphilis.

Because it functions in conjunction with other ingredients, this plant is an essential component of polyherbal medicinal formulations used to treat a variety of diseases. It possesses preventative, immunomodulatory, anti-oxidant, and other properties that aid the body in repairing a variety of diseases. It can also enhance memory and function as a brain tonic in cases of senile and presenile dementia. Anti-spasmodic, anti-oxidant, anti-inflammatory, anti-diabetic, anti-allergic, anti-stress, anti-periodic, anti-arthritic, anti-leprotic, and anti-neoplastic properties of this plant have recently aroused interest. *T. crisp*a has been used in the management of thirst, heat-clearing, and hunger resistance, as well as in the treatment of diabetes [12]. *T. baenzigeri* in traditional medicine is useful in curing antimalarial, diarrhoea, cold, headache, and fever [21], as indicated in Table 1.

**Table 1:** Ethnomedicinal usage of *Tinospora cordifolia* and *Tinospora* species.

Plant Species	Plant Part	Ethnomedicinal Uses	Reference
*T. cordifolia*	Whole plant, root	HIV drug resistance and HIV protease inhibitors. Tyramine is a neuro-modulator used to inhibit neurotransmitters and treat depression and anxiety.	[[22], [23], [24]]
	Stem, root	Anticancer, antiviral.	[22,[25], [26], [27]]
	Whole plant	Antiviral, Anti-inflammatory, Anti-hypertensive, Anti-microbial.	[[28], [29], [30], [31], [32]]
	Stem	Treat neurological impairments, such as those brought on by ALS, Parkinson's disease, dementia, and aberrant movement.	[[33], [34], [35], [36], [37], [38], [39]]
	Stem, aerial part	Osteoporosis and IgA neuropathy are brought on by glucocorticoids in early inflammatory arthritis.	[[40], [41], [42], [43]]
*T. crispa*	Whole plant	Anti-diabetic	[44]
	Stem	Anti-inflammatory	[45]
*T. baenzigeri*	stem	Antimalarial	[21]
*T. sinensis*	Root	Diuretic, immunomodulatory, acute rheumatoid arthritis, Antidepressant and aphrodisiac activity,	[46,47]
	Whole plant	Antimicrobial, anti-inflammatory, Activity of hyperlipidemia	

The *T. sinensis* (Lour.) root powder was used to treat acute rheumatoid arthritis [48], and its main chemical component has been noted for its ability to suppress the development of malignant cells as well as metastasis and angiogenesis. The roots include Fe, K, Mg, and Ni, among other components, which are believed to have significant involvement in the diuretic and aphrodisiac effects. Its roots and leaves show notable action against *Staphylococcus aureus* [46,47]. Ethnomedicinal usage of *Tinospora cordifolia* and *Tinospora* species [[22], [23], [24], [25], [26], [27], [28], [29], [30], [31], [32], [33], [34], [35], [36], [37], [38], [39], [40], [41], [42], [43], [44], [45]] are mentioned in Table 1. There is not enough scientific research documenting the traditional applications of *T. maqsoodiana, T. formanii, T. glabra, T. smilacina*, and *T. subcordata* species within the Indian traditional medicine system.

## 4 Chemical composition

**Fig. 1:**
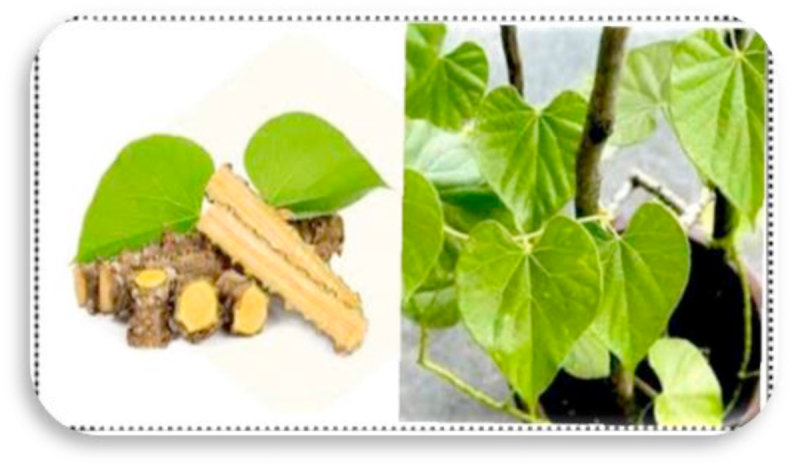
*T. cordifolia*.

**Fig. 2:**
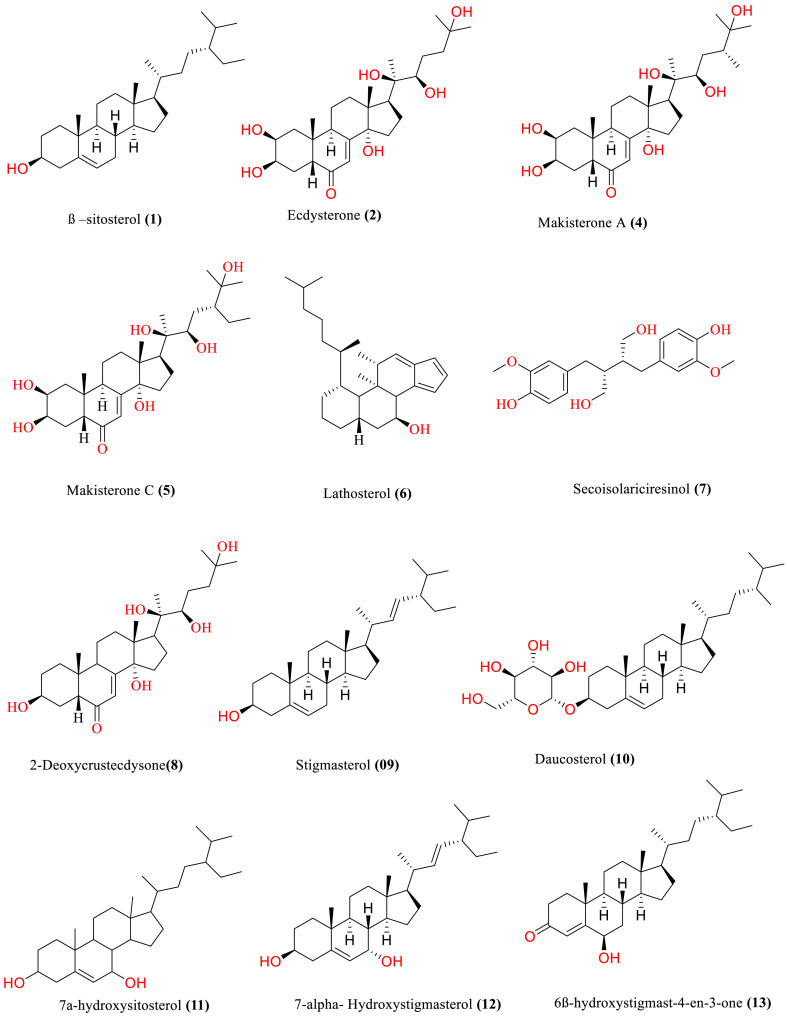
Steroids of *T. cordifolia*, *T. sinensis*.

**Fig. 3:**
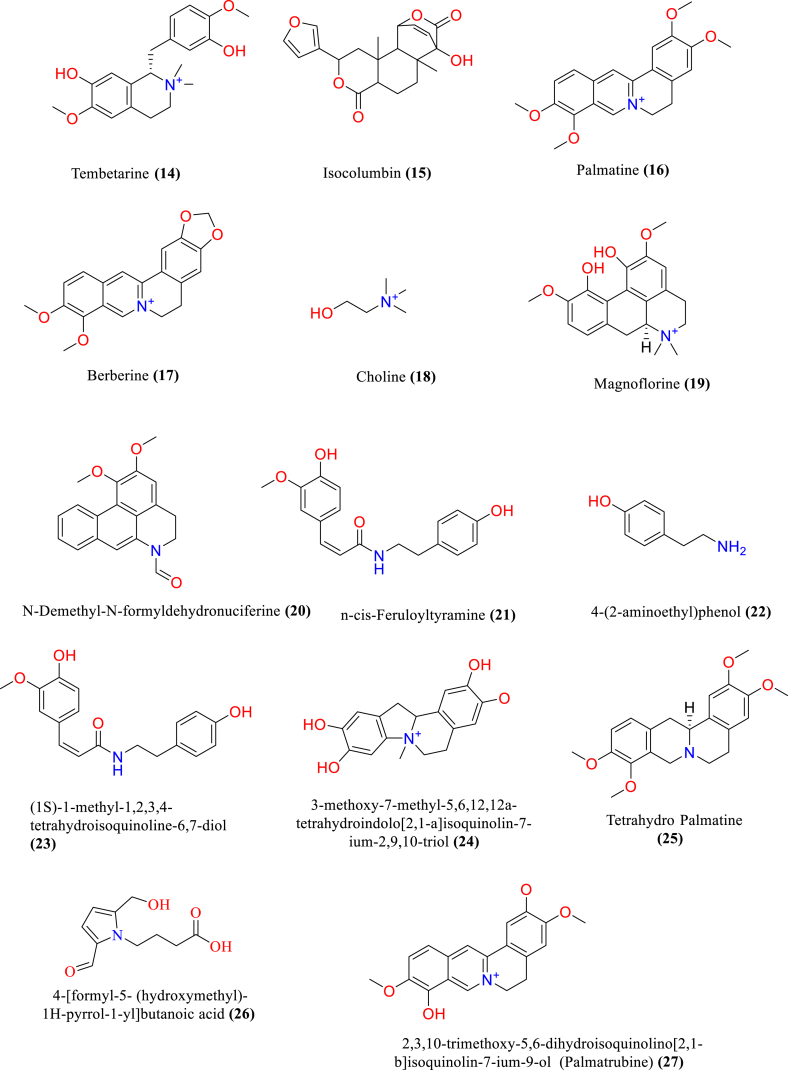
Alkaloids isolated from *T. cordifolia, T. crispa* and *T. sinensis*.

**Fig. 4:**
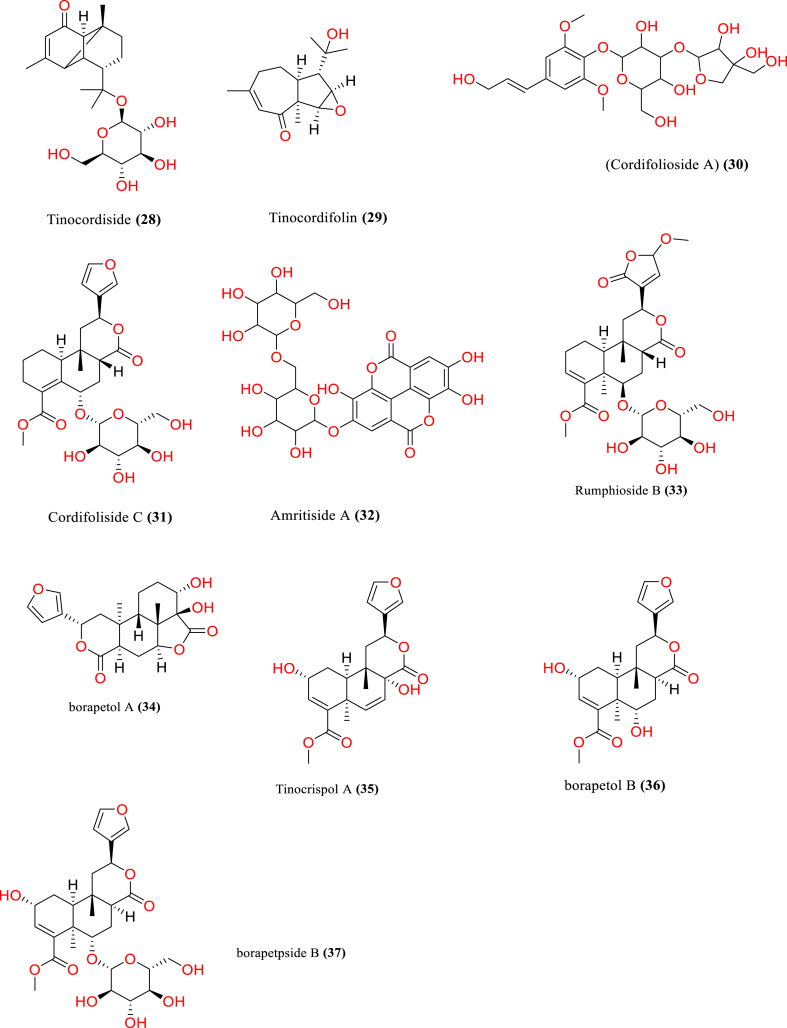

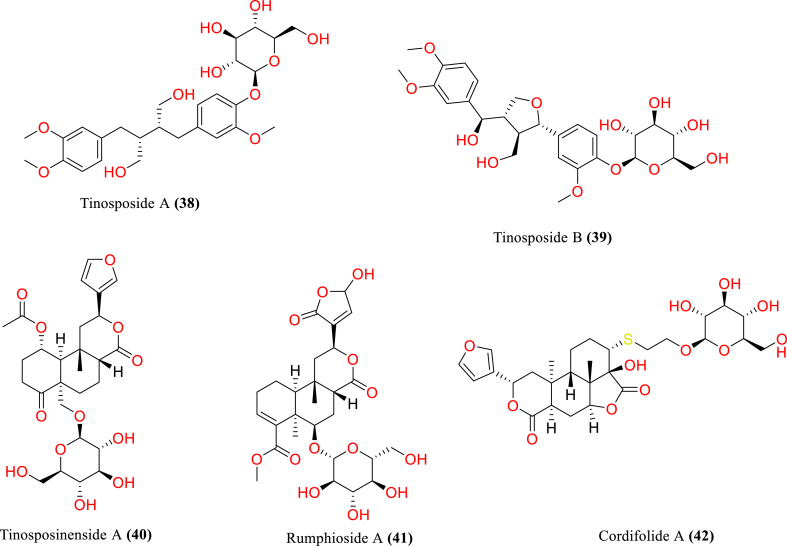
Glycosides of *T. cordifolia* and *T. crispa* and *T. sinensis*.

**Fig. 5:**
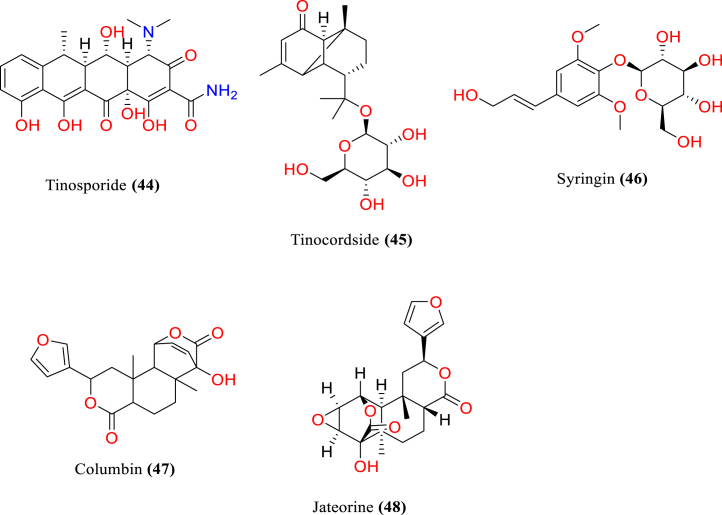
Terpenoids, Diterpenoid lactones and Sesquiterpenoids of *T. cordifolia*.

**Fig. 6:**
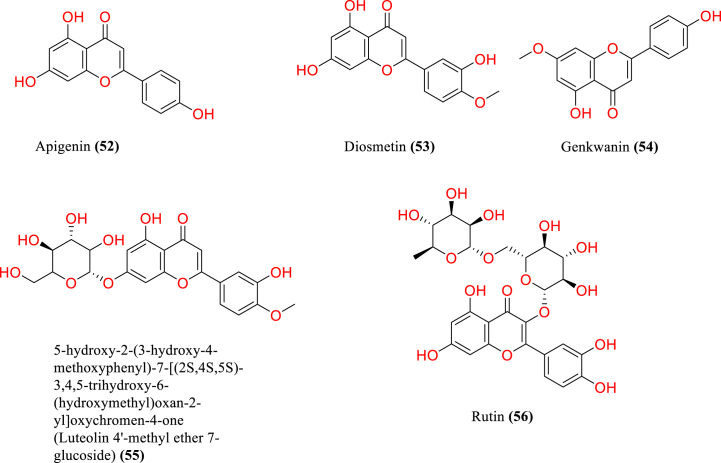
Flavonoids of *T. crispa*.

**Fig. 7:**
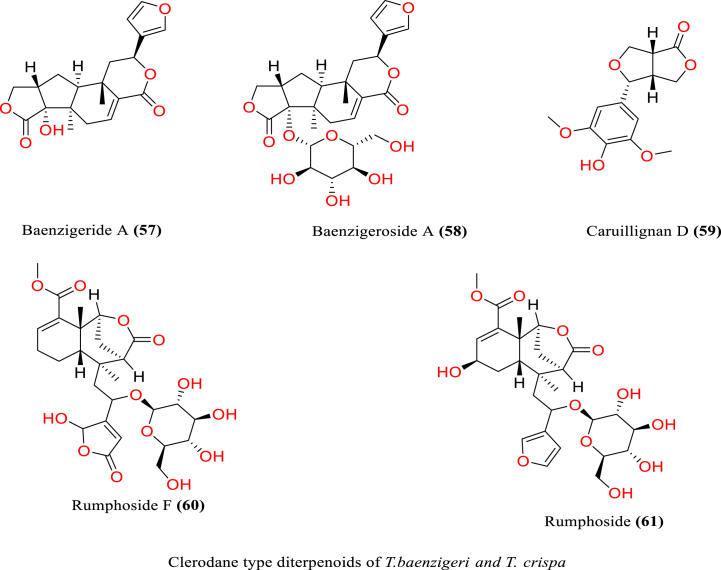
Clerodane type diterpenoids of *T. baenzigeri* and *T. crisp*.

**Fig. 8:**
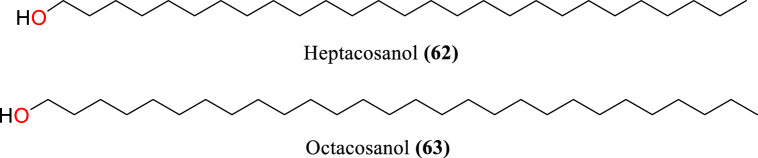
Aliphatic compounds of *T. crispa*.

**Fig. 9:**
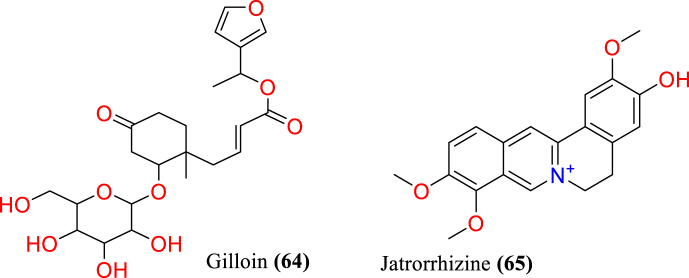
Miscellaneous compounds isolated from *T. cordifolia*.

Table 2 and Fig. 2, Fig. 3, Fig. 4, Fig. 5, Fig. 6, Fig. 7, Fig. 8, Fig. 9 reports the chemical constituents of *T. cordifolia* and three other species viz (see Fig. 1). *T. crispa* (L.), *T. sinensis* (Lour.), *T. baenzigeri* belong to different classes such as glycosides, alkaloids, terpenoids, steroids, polysaccharides, sesquiterpenoids [49], etc. The main phytochemicals in *T. cordifolia* include tinosporide, tinosporine, tinosporaside, cordifol, cordifolide, diterpenoid furanolactone, clerodane furano diterpene, tinosporidine, columbine, and b-sitosterol [[49], [50], [51]]. The rest of five species have not been investigated for the presence of phytochemicals. No compound is reported from 5 Indian *Tinospora* species (*T. glabra, T. formanii, T. smilacina, T. maqsoodiana, and T. subcordata*).

**Table 2:** Isolated Chemical constituents and mechanism of action of Indian *Tinospora cordifolia* and *Tinospora* species.

Plant Species	Sr.no.	Isolated Compounds	Active Component	Plant parts	Mechanism of action	References
*T. cordifolia*	1.	β-sitosterol	Steroids	Stem	Decrease inflammation	[41]
	2.	Ecdysterone				
	3.	Giloinsterol				
	4.	Makisterone A				
*T. crispa*	5.	Makisterone C		Stems, aerial parts, vines stem	Remarkable α-glucosidase and α-amylase inhibition.	[52,53]
	6.	Lathosterol				
	7.	Secoisolariciresinol				
	8.	2-Deoxy crustecdysone				
	9.	Stigmasterol		Root	Reduce inflammation	
*T. sinensis*	10.	daucosterol		Stem	Anti-neuro-inflammation, regulation of immunity	[48,54,55]
	11.	7α-hydroxysitisterol				
	12.	7α-hydroxy Stigmasterol				
	13.	6β-hydroxystigmast-4-en-3-one				
*T. cordifolia*	14.	Tembetarine	Alkaloids	Root, stem	Anti-histamine, CNS Stimulant.	[22,25,26,56]
	15.	Isocolumbin				
	16.	Palmetine				
	17.	Berberine				
	18.	Choline				
	19.	Magnoflorine				
*T.crispa*	20.	N-Demethyl-N-formyldehydro-nuciferine		Stem	Important immune-modulatory effects include increased phagocytic activity, chemotaxis, ROS and NO generation, and TNF-α,IL-1β, IL6, PEG2, and MCP-1 production.	[52,57,58]
	21.	N-cis-feruloyltyramine				
	22.	4-(2-aminoethyl) phenol (Tyramine)			Substantial antioxidant activity and effects of positive inotropy on the isolated left atrium.	
	23.	(1S)-1-methyl-1,2,3,4-tetrahydro-isoquinoline-6,7-diol (Salsolinol)				
	24.	3-methoxy-7-methyl-5,6,12,12a-tetrahydro indolo [2,1-a] iso-quinolin-7-ium-2, 9,10-triol (-litcubinine)				
*T. sinensis*	25.	Tetrahydro palmatine		Stem	Antiviral action	[48,54]
	26.	4-[formyl-5-(hydroxyl-methyl)-1H-pyrrol-1-yl] butanoic acid				
	27.	2,3,10- trimethoxy-5,6-dihydro-iso-quinolino [2,1-b] isoquinolin-7-ium-9-ol (Palmatrubine)				
*T.cordifolia*	28.	Tinocordiside	Glycosides	Stem	Prevent cardiac Na+, K + ATPase activity	[33,34,36,37,39,59,60]
	29.	Tinocordifolin				
	30.	Cordifolioside A				
	31.	Cordifolioside C				
	32.	Amritiside A				
*T. crispa*	33.	Rumphioside B		Stem, whole plant	Immunomodulatory action	[[61], [62], [63]]
	34.	Borapetol A				
	35.	Tinocrispol A,				
	36.	Borapetol B				
	37.	Borapetpside B				
*T. sinensis*	38.	Tinosposide A		Stem	Antioxidant activity	[64,65]
	39.	Tinosposide B				
	40.	Tinosposinenside A				
	41.	Rumphioside A				
	42.	Cordifolide A				
	43.	Furanolactone				
*T. cordifolia*	44.	Tinosporide	Diterpenoid Lactones and sesquiterpenoids	Whole plant	Cardio-protective	[28,[30], [31], [32],^66^]
	45.	Tinocordside				
	46.	Syringin				
	47.	Columbin				
	48.	Jateorine				
	49.	14-dieno-17, 12S: 18, 1S dilactone],				
	50.	Tinosporon				
	51.	Clerodane derivatives [(5R,10R)-4R-8Rdi-hydroxy-2S-3R: 15, 16- diepoxy-cleroda-13				
*T. crispa*	52.	Apigenin	Flavonoids	Stem	Cardio-protective	[67]
	53.	Diosmetin				
	54.	Genkwanin				
	55.	Luteolin 4′-methyl ether 7-glucoside				
	56.	Rutin				
*T. baenzigeri*	57.	Baenzigeride A	Clerodane-type furano diterpenoids and diterpenoids	Stem	Diarrhoea, cold, fever and ulcers	[68,69]
	58.	Baenzigeroside A				
	59.	Caruillignan D				
*T. crispa*	60.	Rumphioside F		Stem aerial parts	Substantial cytotoxicity against MDA-MB 231 breast cancer cells that are STAT3-dependent	[70]
	61.	Rumphoside				
*T. cordifolia*	62.	Heptacosanol	Aliphatic compounds	Whole plant	–	[23]
	63.	Octacosanol				
	64.	Gilloin	Miscellaneous			
	65.	Jatrorrhizine

### 4.1 Phenolics

Four phenyl propanoids [[71], [72], [73]], two flavonoids [38,74], three lignans [72,73], and two benzenoid derivatives [75,76] have been discovered as phenolics.

### 4.2 Alkaloids

The alkaloids in a plant are a crucial secondary metabolite. The protoberberine alkaloids palmatine, berberine, magnoflorine, jatrorrhizine, and corydine [[77], [78], [79]] are among the thirteen alkaloids having aporphine and isoquinoline skeletons, as well as amide, and amine that have been discovered.

### 4.3 Terpenoids

*T. cordifolia* produced one monoterpene [72], five sesquiterpenoids [8,[79], [80], [81]], 32 diterpenoids and their glycosides with norclerodane and clerodane skeletons, as well as one triterpenoid called cycloeuphordenol [79]. The whole plant was used to get the bicyclic diterpenoid tinosporin.

### 4.4 Steroids

In addition to sitosterol, four steroids [[82], [83], [84], [85]] and 2, 3, 14, 20, 22, 25 -hexahydroxyl-5-cholest-7-en-6-one have also been discovered.

### 4.5 Aliphatic chemicals and essential oils

The essential oil extracted from fresh leaves using hydrodistillation contained various compounds, including phenols (16.6 %), alcohols (32.1 %), fatty acids (15.7 %), aldehydes (16.2 %), esters (3.2 %), alkanes (8.3 %), and terpenes (1.2 %) [86]. Additionally, the GC-MS analysis of a hexane extract of stems revealed the presence of hydroquinone (16.6 %), palmitic acid (14.1 %), 2-hexenal (14.2 %), phytol (11.4 %) ethyl-9,12-octadecadienoate, methyl 9-octadecenoate, methyl hexadecanoate, and methyl octadecanoate [87].

## 5 Pharmacological action

*T. cordifolia* has long been recognized as the predominant plant utilised in traditional medicine throughout history. The plant possesses numerous beneficial attributes and exerts a substantial impact on the defence system. The stem is employed as a bitter tonic for the stomach and as a laxative, while the root is utilised for stress relief and as an antimalarial agent [88], suggests that it enhances bile synthesis, improves blood quality, and alleviates jaundice. A lot of research has been done on the drug guduchi to find out how well it might work as a medicine. It improves several body systems. Ayurveda considers it a Rasayana with global effects [89,90]. Several research has reported various pharmacological effects of *T. cordifolia* and other Indian *Tinospora* species, which are covered in this article.

### 5.1 Anti-diabetic property

The stem of *T. cordifolia* is frequently used in traditional Indian medicine to treat diabetes by regulating blood glucose levels. According to reports, it possesses anti-diabetic properties that regulate blood sugar by increasing insulin secretion, reducing oxidative stress (OS), reducing gluconeogenesis, and reducing glycogenolysis. *T. cordifolia* contains anti-diabetic tannins, alkaloids, flavonoids, cardiac glycosides, saponins, and steroids as its primary phytoconstituents [[91], [92], [93]]. Isoquinoline alkaloid-rich stem fraction, including jatrorrhizine, palmatine, and magnoflorine, has been demonstrated to mimic and release insulin during *in-vitro* and *in-vivo* investigations. It has been shown that ingesting root extracts can reduce blood sugar levels, increase insulin production, and prevent OS symptoms. *In-vitro* studies demonstrated the activation and restoration of glutathione peroxidase (GPx), superoxide dismutase (SOD), and glutathione (GSH), as well as the inhibition of glucose 6-phosphatase and fructose 1, 6-diphosphatase, and the liver's glycogen content. The crude stem extracts of *T. cordifolia* in chloroforms, ethyl acetate, dichloromethane (DCM), and hexane inhibited salivary and pancreatic amylase and glycosidase, which increased postprandial glucose levels and may be useful in the treatment of diabetes mellitus [94].

The administration of *T. cordifolia* root extract to diabetic rats resulted in a reduction in their blood levels of glycosylated haemoglobin, hydroperoxides, ceruloplasmin, and vitamin E. Oral use of *T. cordifolia* extract has been shown to decrease blood levels of GSH and vitamin C [95]. Additionally, the Ayurvedic herbal composition “Ilogen Excel” consists of eight separate medicinal herbs. These include *Curcuma longa, Salacia oblonga, Strychnos potatorum, Tinospora cordifolia, Coscinium fenestratum, Vetivelia zizanioides,* and *Andrographis paniculate*. GSH, GPx, and SOD levels and catalytic activity are reduced in the hearts and brains of diabetic rodents [96]. TCE(Trichloroethylene) increases body weight, total hemoglobin, and hepatic hexokinase in diabetic rats while decreasing hepatic glucose-6-phosphatase, serum acid phosphates (ACP), alkaline phosphates (ALP), and lactate dehydrogenase (LDH) [97]. TCE was discovered to be protective in the presence of higher antioxidant molecule and enzyme concentrations. TCE has been shown to substantially reduce diabetes-related oxidative stress in the maternal liver by decreasing malondialdehyde and reactive oxygen species (ROS) and increasing GSH and total thiols [98].

In patients with low to severe hyperglycemia, the aqueous extract of *T. cordifolia* significantly decreased blood sugar levels. The group receiving 400 mg kg per day for moderate diabetes had the greatest decrease in glucose concentration by percentage. In experiments lasting 21–120 days, Alloxan rats served as test subjects. Significant glycemic control and an effect on key metabolic enzymes implicated in glucose metabolism in diabetic patients with mild to severe hyperglycemia. Body mass, total hemoglobin, and hepatic hexokinase concentrations all increase [99]. Streptozotocin rats exposed to aqueous *T. cordifolia* extract for six weeks exhibited significant anti-hyperglycemic activity a substantial decrease in blood and urine glucose levels [100].

*T. cordifolia* extracts have demonstrated anti-diabetic effects in vivo, according to pharmacological investigations. When the aqueous extract of the stem of another *Tinospora* species, “*Tinospora crispa*," was examined, anti-hyperglycemic properties were found. This is likely due to the fact that it stimulates insulin secretion by regulating cell and Ca2+ concentration [92]. Borapetoside C from *Tinospora crispa* (5 mg/kg, i. p.) decreased elevated plasma glucose levels, increased glucose intake, delayed the onset of insulin resistance, and ultimately improved insulin sensitivity in diabetic rats. Borapetaside C may have had a hypoglycemic effect due to the stimulation of insulin-induced IR-Akt-GLUT2 expression in the liver and its improvement of insulin sensitivity [101].

### 5.2 Antioxidant activity

The plant's capacity to enhance cell strength is derived from a polysaccharide known as arabinogalactan and a phenolic compound called epicatechin [74,102]. The leaf extract powder of the plant has higher antioxidant capacities compared to its stem extract powder [103]. The alkaloid components of this plant's root extract include antioxidant capabilities, which provide protection against nephrotoxicity caused by aflatoxin [56].

*T. cordifolia* root extracts can help replenish cell-reinforcement pointers like GPx, SOD, and GSH [22]. In the maternal livers of diabetic rats, *T. cordifolia* extracts have been demonstrated to decrease reactive oxygen species (ROS) and malondialdehyde while raising GSH levels [20,104].

### 5.3 Antimicrobial activity

*Salmonella paratyphi, Staphylococcus aureus, Escherichia coli, Proteus vulgaris, Klebsiella pneumoniae, Salmonella flexneri, Salmonella typhimurium, Enterobacter aerogene, Pseudomonas aeruginosa,* and *Serratia marcesenses* (Gram-positive bacteria) were tested for antibacterial activity against *T. cordifolia* extracts [105]. Extractions from *T. cordifolia* water, ethanol, and acetone are used to hook leaves and roots. In clinical isolates of urinary pathogens, F. Thoms demonstrated the strongest inhibitory efficacy against *Klebsiella pneumoniae* and *Pseudomonas aeruginosa* [106] strains obtained from burn victims show very great antibacterial activity when treated with silver nanoparticles made from the *T. cordifolia* stem [107]. It was shown that the active ingredient in *T. cordifolia* stem ethanol extracts [(5R, 10R)-4R, 8R-Dihydroxy-2S, 3R:15, 16-diepoxyclerod-13(16), 17, 12S, 18, 1S-dilactone] is effective against both bacteria and fungi. The lowest MIC values were found for *Bacillus subtilis* (200 g/ml) and *Enterococcus faecalis* (125 g/ml). The substance was also effective against fungi, with the lowest MIC values for *Trichophyton rubrum* 57 (62.5 g/ml), *Trichophyton simii* (31.25 g/ml), and *Trichophyton rubrum* 296 (62.5 g/ml) [108]. The components in *T. cordifolia* exhibited a greater inhibitory effect against reference microbiological strains and clinical isolates of methicillin-resistant Staphylococcus aureus (MRSA) and Klebsiella pneumoniae-developing carbapenemase, according to the findings of the Francesca Bonvicinia et al. investigation [109].

### 5.4 Antiulcer activity

Researchers tested the antiulcer effect of the roots using ethanolic extracts. It has been shown that *T. cordifolia* has a potent, diazepam-like preventive effect against ulceration caused by 8-h restriction tension [87,110].

### 5.5 Anticancer action

In mice, berberine exhibits anti-cancer properties. Ehrlich inhibits topoisomerase II at a dose of 10 mg/kg body weight in ascites carcinoma [[111], [112], [113]] [[111], [112], [113]] [[111], [112], [113]]. Columbin, a furanolactone diterpenoid, on the other hand, has demonstrated chemopreventive effectiveness against human colon cancer [28]. Octacosanol, long-chain aliphatic alcohol, inhibits MMP activity, NF kappa B translocation to the nucleus, and vascular endothelial growth factor synthesis by cancer cells into ascites fluid (*in-vivo*) [114]. By raising GSH, SOD, and catalase levels and reducing DNA damage, the plant alkaloid palmatine can reduce tumour size [115]. G1-4A can stimulate cytotoxic T lymphocytes that can destroy cancer cells by activating dendritic cells generated from bone marrow [116,117] By decreasing the population of drug-resistant cancer cells (rich in ATP-binding cassette transporters), its ethanolic extract might assist chemotherapy in overcoming such difficulties in cancer therapy [118]. Humans with MCF-7 breast cancer and chemically produced liver cell carcinoma can both be protected by ECD discovered in *T. cordifolia* [30,119]. ECD controls the expression of p53, Cdkn2A, and the mdm2 gene in cancer cells, leading to their demise [120]. This plant's octacosanol is an antiangiogenic drug that inhibits tumour spread and growth. It can increase pro-apoptosis and senescence while inhibiting mechanisms that reverse apoptosis, which has a therapeutic effect on neuroblastoma [121]. The capacity of this plant's extract to lessen CYP3A4, a crucial enzyme in the metabolism of chemotherapy drugs, makes it suitable for usage as an adjuvant with chemotherapy drugs. This might diminish the harmful effects that could otherwise have an influence on cells under normal circumstances and lower the number of hazardous drugs, such as those used to treat cancer [122]. By regulating pro-inflammatory cytokines and GSH like TNF, it also lessens the toxicity of anti-cancer drugs. A trademark has been filed for an eleven-part herbal remedy for the treatment of cancer that comprises 17 to 23 percent *T. cordifolia*. The hemoptysis and chest discomfort in a patient [123]with pulmonary epidermoid carcinomas (who refused previous therapies) completely stopped after one month of treatment with 450–480 mg of gelatinous capsule form TDS. Additionally, the patient's appetite had improved. When used as a tumoristatic drug, the same formulation was successful in treating a patient with third-stage pulmonary epidermoid carcinomas who had not responded to prior treatments [20].

### 5.6 Hepatoprotective activity

*T. cordifolia* has traditionally been recommended for reducing hepatosplenomegaly, stimulating bile flow, and treating Kamala (jaundice) in cases of obstructive lesions. Over 33 % of the hepatoprotective formulas available in the Indian market contain this substance, and it can also be consumed independently as a standalone agent [124]. Instead of inducing hepatotoxicity, this plant is crucial for its hepatoprotective properties [125]. In cases of carbon tetrachloride-induced liver injury, *T. cordifolia* has been seen to preserve normal metabolism and lower levels of particular enzymes such aspartate, alanine aminotransferase (ALT), alkaline phosphatase (ALP), aminotransferase (AST) and total bilirubin. The powerful hepatoprotective activities of *T. cordifolia* extract can be related to its ability to stimulate liver regeneration, as well as its antioxidant and free radical-scavenging properties [124,126] [[124], [126], [127], [128]] [[124], [126], [127], [128]]. A standardised aqueous extract was used to study patients with hepatic disorders who showed signs of immunosuppression and/or fibrosis. The most thorough clinical research was done on patients with obstructive jaundice, where it was discovered that adding *T. cordifolia* (16 mg/kg/day) to conventional therapy prior to surgical correction significantly decreased mortality, dropping it from 61.54 % to 25 % in patients with PTBD (percutaneous transhepatic biliary drainage) and from 39 % to 6.25 % in patients without PTBD. This was related to fewer individuals in the *T. cordifolia* treated group experiencing septicemia [129,130]. At a dosage of 200 mg/kg, *T. sinensis* significantly decreased paracetamol-induced elevated levels of serum ALT, AST, ALP, and bilirubin, displaying normal liver architecture and indicating a hepatoprotective potential [131].

### 5.7 Immunomodulator activities

Isolated chemical substances including cordifolioside A and guduchi syringin were referred to be immunomodulators in the clinical examination [8]. The stem of *T. cordifolia* influences the quantity of enzymes like catalase and stimulates lymphocyte cells, emphasizing the immune protective action of this shrub [132]. *T. cordifolia* extract causes macrophage cells to create more myeloperoxidase and other enzymes, which enhances antibacterial activity and increases immunity [133]. On the other hand, it increases the phagocytic activity of macrophages. Additionally, it stimulates macrophages and splenocytes, because there is a higher level of nitric oxide produced, which suggests anti-tumor and immune protective effect [134]. One research investigation found that *T. cordifolia* lotion lowers interleukin-1 and IL-6 levels in an animal model of scabies. Its anti-scabies effectiveness is demonstrated by the suppression of hyperkeratosis and inflammatory cell infiltration into scabietic wounds [135]. Aqueous extract induces cellular mitosis and increases the generation and activation of immune effector cells and cytokine.

*T. cordifolia* is a potent preventative for diseases with an immune system component since it may also enhance neutrophil and immune cell activity [136]. Alkaloids, steroids, aliphatic chemicals, and other guduchi substances showed a significant immuno protective effect when tested in a preclinical rat model. The polysaccharide G1-4A, which is generated from the *T. cordifolia* plant, stimulates the growth and development of T and B immune cells as well as the production of the anti-apoptotic gene [137]. It has been shown that the chemical D-glucan generated from TC maintains body physiology by stimulating lymphocyte cells [138]. *T. cordifolia* extracts caused PMN cells to undergo phagocytosis. Increases in bone marrow cells, white blood cell (WBC) count, and foot pad thickness are all brought on by *T. cordifolia* alcoholic extract (100 mg/kg), showing a stimulatory effect on the haemopoetic system with a strong immunomodulatory effect [139]. A traditional Ayurvedic preparation of *T. cordifolia's* aqueous extract known as “Ghana” reduced the edematogenic agents when tested on the edoema rat model, and as a result, exhibited potent immunostimulatory properties [97].

### 5.8 Antitoxic properties

As stated in literature [140], *T. cordifolia* possesses the ability to eliminate harmful free radicals and provides a protective effect on the body by modulating hormone and mineral levels. Evidence shows that *T. cordifolia* effectively reduces reactive oxygen species (ROS) and enhances hormone levels (such as glutathione) and enzyme activity (such as catalase and glutathione reductase) in the kidneys of Swiss albino mice. The presence of alkaloids in this plant is responsible for its inherent anti-toxin capabilities. *T. cordifolia* has shown efficacy in preventing damage and exhibiting its antitoxin activity in the urinary bladder and hepatic cells by significantly elevating the level of reduced glutathione content and cytokines, while gradually decreasing inflammatory cytokines (Tumour necrosis factor) [123]. *T. cordifolia* safeguards the kidneys by enhancing the activity of antioxidant enzymes, ascorbic acid, protein, and glutathione (GSH) [141].

### 5.9 Anti-HIV activity

The usefulness of *T. cordifolia* in treating HIV-positive patients by reducing resistance to the retroviral therapy has been investigated [142]. The anti-HIV action of *T. cordifolia* in HIV-positive patients increases CD4 T-cell count and decreases eosinophil count, demonstrating its usefulness in disease treatment. The *T. cordifolia* extract significantly improved the phagocytic and intracellular bactericidal activities. Activation of peritoneal macrophages was another effect of *T. cordifolia*. It also boosts intracellular killing and phagocytosis. B-lymphocytes, polymorph nuclear leucocytes, and macrophages are all significantly activated by *T. cordifolia* [[143], [144], [145]].

### 5.10 Antipyretic properties

A 95 % ethanolic extract of *T. cordifolia* included a water-soluble component that was discovered to be antipyretic. Another study found that *T. cordifolia* stems exhibit antipyretic properties in the hexane and chloroform soluble parts. *T. cordifolia* has anti-infective and antipyretic effects, according to several research. Before being treated with *T. cordifolia*, rats didn't die from intra-abdominal sepsis after having their cecum tied, and it greatly reduced death rates in mice from *E. coli* caused peritonitis [146].

### 5.11 Nephroprotective properties

*T. cordifolia's* role in nephrotic syndrome (NS) shows that it contains antioxidant, immunomodulating, anti-inflammatory, and nephroprotective qualities that can treat nephritis. The effects of steroids and NS rebound are rare. *T. cordifolia* improves novel medication efficacy and safety. It can also be used with modern drugs to treat steroid-resistant and steroid-dependent Nephritic Syndrome [132,147]. Table 3 presents the main species, namely *T. cordifolia, T. crispa* (L.), and *T. sinensis* (Lour.), which have been demonstrated by researchers [[148], [149], [150], [151], [152], [153], [154], [155], [156], [157], [158], [159], [160], [161], [162], [163], [164], [165], [166], [167], [168], [169], [170], [171], [172], [173], [174], [175]] to possess significant medicinal properties.

**Table 3:** Pharmacological activities of *Tinospora cordifolia* and other *Tinospora* species.

S. No	Activity	Part/Extract	Species	Animal Model/Cell Lines	Result	Reference
1.	Analgesic activity	Whole plant/Alcohol extract C	*T. cordifolia*	Albino rats were subjected to abdominal writhing and a hot plate.	The anti-nociceptive activity was evaluated by the latency times.	[148]
			2. *T. crispa*			
			3. *T. sinensis*	Tests on adult male Wistar rats using the tail flick and acetic acid to cause writhing/p.o.	Significant (P < 0.001) increase in latency time was seen in the extract.	[47]
4.	Antidyslipidemic activity	Stem extract	*T. cordifolia*	Charles Foster strain adult male rats who have developed diabetes due to alloxan.	As a natural remedy*, T. cordifolia* is more effective at reversing diabetic dyslipidemia and oxidative stress. Treatment had a positive impact on HDL synthesis, which may have helped to control lipid metabolism.	[149]
			5. *T. crispa*	Male with alloxan diabetes model Wistar albino rats	The compound increased serum insulin levels while lowering fasting blood glucose levels.	[150]
		6. Flower and Leaf extract	*T. sinensis*	Male Wistar rats were given hydrogenated groundnut oil to produce hypercholesterolemia using p.o.	Extract changed the inappropriate metabolic profile and significantly increased the risk of hypolipidemia (P 0.05).	[46]
7.	Immunomodulatory activity	ethanol and aqueous extract from the whole plant	*T. cordifolia*	Swiss male albino mice.	*T. cordifolia* stem contains strong immunomodulatory properties, and these properties may be a result of the drug's individual chemical components or their combined effects.	[132]
		8. Aqueous	*T. sinensis*	Anemia brought on by cyclophosphamide in male Wistar rats, p.o.	Extract shown a considerable effectiveness.	[125]
		9. Fraction and ethanolic	*T. crispa*	MTT proliferation test and intracellular cytokine analysis in LPS-stimulated murine macrophage cell line RAW264.7.	RAW264.7 cell viability and the intracellular expression of the cytokines INF-c, IL-6, and IL-8 were increased by the extract and fractions.	[151]
10.	Cardioprotective effect	Extract of whole plants	*T. cordifolia* *T. sinensis* *T. crispa*	Intravenous calcium chloride administration causes arrhythmia in rats	Strong evidence points to a potential function for *T. cordifolia* in cancer chemoprevention as a result of the therapy with *Tinospora*, which caused a significant induction in the particular activities of detoxifying enzymes in the kidney.	[56]
11.	Aphrodisiac property	Hydroalcoholic and aqueous and extract	*T. cordifolia*	Adult albino rats of wistar strain.	In order to assess the potential mechanism of the medicine, it was recommended that in the future, the components in the hydro-alcoholic extract that are responsible for the aphrodisiac activity be isolated and their aphrodisiac potential be tested in both in-vitro and in-vivo models.	[152]
12.	Antidiarrheal activity	Whole plant/Aqueous extract	*T. cordifolia* *T. sinensis* *T. crispa*	Magnesium sulphate and castor oil induced diarrhoea in albino rats	The outcomes established pharmacological support for the traditional uses of *T. cordifolia* as an antidiarrheal and antiulcer drug.	[110]
13.	Antiulcer activity	Ethanol, aqueous, and whole plant extracts	*T. cordifolia,* *T. sinensis*	Pylorus ligation-induced ulcer in albino rats		
14.	Neuroprotective effect	Aerial parts/Ethanol extracts	*T. cordifolia*	Parkinson's disease rat models with 6-hydroxydopamine lesions.	The mitochondrial activity that TCEE was able to retain offered a potentially effective method for treating clinical PD. To put it to use in clinical researches, additional pharmacological and clinical studies are required.	[153]
15.	Anti-inflammatory activity	Stem/Aqueous extract	*T. cordifolia*	Rat paw edema model caused by carrageenan.	Paw volume was significantly reduced by extract (P 0.05).	[97,154]
		16. Whole plant/Diosgenin	*T. sinensis*		In comparison to indomethacin, diosgenin exhibited the most pronounced anti-inflammatory effect, with a percentage of 82.25 %. This effect was significant (P < 0.01), as it substantially reduced the average volume of paw edoema 3 h after the administration of carrageenan.	[155]
		17. Stem/aqueous and methanolic	*T. crispa*	Inflammation was triggered by TNF-a in human umbilical vein endothelial cells.	Significant signaling molecule reduction was seen in both extracts for ICAM-1, VCAM-1, MCP-1, and M-CSF.	[156]
18.	Gastroprotective activity	Whole plant	*T. cordifolia*	In rats, plant indomethacin caused stomach ulcers.	Based on our findings, we hypothesised that the ECD (Epoxy clerodane diterpene) substance found in *T. cordifolia* may be to blame. Due of ECD's strong biological activity, further research is appropriate to be done in order to turn it into a pharmaceutical.	[157]
19.	Antioxidant activity	Whole plant/Ethanol extract	*T. cordifolia*	In male wistar albino rats, N-nitrosodiethylamine caused liver cancer.	*T. cordifolia* treatment of group III *T. cordifolia* treated mice considerably lowers the peroxidation reaction (P 0.01).	[158]
			20. *T. sinensis*	In vitro tests for DPPH, lipid peroxidation, DMSO, ABTS, NO radical scavenging activity, and SOD scavenging.	In vitro experiments using the following extracts revealed considerable antioxidant activity: ABST (IC50 = 90.44 0.36 lg/mL); DPPH (IC50 = 94.66 0.049 lg/mL); DMSO (IC50 = 97.99 0.15 lg/mL); SOD (IC50 = 93.72 0; NO (IC50 = 87.25 2.72 lg/mL).	[159]
			21. *T. crispa*	Free radical scavenging test using DPPH	Methanol extract considerably boosted vitamin E-like radical activity (100 %) and radical scavenging activity (IC50 12 mg/mL).	[158]
22.	Radio protective and Cytoprotective activity	Stem/Ethanol extract	*T. cordifolia*	Genotoxicity was brought on by 4 Gy radiation and cyclophosphamide in albino mice.	Cordifolioside-A, which has radioprotective and cytoprotective activity, is present in the n-butanol fraction of *T. cordifolia* stem extract; however, the precise mechanism underlying these effects is yet unknown and requires further research.	[160]
23.	Antifeedant activity	Whole plant/Chloroform Extract	*T. cordifolia*	*Earias vitella*, *Plutella xylostella*, and *Spodoptera litura* were the microorganisms used.	The most efficient diterpene tested was 8-hydroxy tinosporide 3. Only a small variation existed between the insects' susceptibilities to the diterpenoids, with *S. litura* being slightly more vulnerable than *E. vitella*. Compound 3's antifeedant efficacy at 5 and 10 mg doses was comparable to azadirachtin-A's antifeedant activity at 0.5 mg concentration.	[161]
24.	Ameliorative effect	Root/Ethanol extract	*T. cordifolia*	Swiss male albino mice were exposed to aflatoxin B1.	Strong evidence points to a potential function for *T. cordifolia* in cancer chemoprevention as a result of the therapy with *Tinospora*, which caused a significant induction in the particular activities of detoxifying enzymes in the kidney.	[56]
25.	Hepatoprotective activity	Whole plant/Aqueous Extract	*T. cordifolia*	Rats with bile duct ligation developed jaundice.	The outcomes allowed us to advise against using melatonin and *T. cordifolia* to lessen oxidative stress associated with cholestasis in humans.	[162]
		26. Root/Ethanol	*T. sinensis*	Wistar albino rats with p.o. induced liver damage from CCl4.	Extract decreased sinusoidal dilation and minor inflammatory factors. Serum enzyme levels were found to have significantly decreased (P < 0.001) in treated mice.	[163]
27.	Nootropic effect	Whole plant/Ethanol extract	*T. cordifolia*	Amnesic rats accomplish tasks in the radial arm maze and the Barnes maze test.	It was determined that combining the ethanolic extracts of *Evolvulus alsinoides, Bacopa monnieri,* and *T. cordifolia* produced a stronger nootropic effect.	[164]
28.	Hypoglycemic activity	Stem/Aqueous Extract	*T. cordifolia*	Rat pancreatic β-cell lines were used in vitro to assess the impact of insulin secreted.	The TC's alkaloid content contributed to its antihyperglycemic effects. The postprandial hyperglycemia is improved by isoquinoline alkaloid rich fraction (AFTC), which may have hypoglycemic effects through mechanisms of insulin releasing and insulin-mimicking activities.	[22]
29.	Antipsychotic activity	Aqueous and Ethanol extract	*T. cordifolia*	Mouse model was hampered by amphetamine.	Based on the findings, it can be said that *T. cordifolia* aqueous ethanolic extract has no psychotropic potential at the dose levels tested.	[165]
30.	Antidepressant activity	Stem, fresh leaves/Aqueous and Petroleum ether extract	*T. cordifolia*	The activity of Swiss albino mice was measured with the tail suspension test and the forced swim test.	The serotonin, monoamines noradrenaline, and dopamine were increased, while GABA was decreased, making *T. cordifolia* petroleum ether extract work in a manner similar to an antidepressant.	[166]
			*T. sinensis*		After the first, second, and third weeks of treatment, the extract significantly raised anoxia stress tolerance (P 0.01); it also decreased high levels of serum biochemical parameters and blood cell count and avoided changes in the liver and adrenal gland weight.	[131]
31.	Anti osteoporotic activity	Stem/Ethanol extract	*T. cordifolia*	Sprague-Dawley female rats.	However, reproductive organs like the uterus and mammary gland did not exhibit estrogen-like actions from TC extract. As a result, our study shows that *T. cordifolia* extract has the potential to be employed as an antiosteoporotic drug.	[167]
32.	Antineoplastic activity	Aerial parts/DCM extract	*T. cordifolia*	Transplants of Ehrlich ascites carcinoma in mice.	The TCE is an amalgam of several alkaloids, including berberine. The combination of other alkaloids and TCE's anti-cancer effects may have an impact. Optimistic outcomes from pre-clinical and clinical tests on this extract and its other components suggest that they could become a potent cancer therapy tool and a standard component of cancer treatment procedures.	[111]
33.	Antifertility effect	Methanol extract	*T. cordifolia*	Male rats.	Accordingly, the findings imply that oral administration of a crude methanolic extract of *T. cordifolia* stem can cause male rats to become infertile as a result of interfering with the levels of androgen in the testes, which changes the process of spermatogenesis.	[168]
34.	Antiasthamatic activity	Stem/Hydroalcoholic Extract	*T. cordifolia*	In an in vivo asthma model, mice were sensitized by intraperitoneal and intranasal administration of ovalbumin.	The *T. cordifolia* extract exhibits therapeutic potential for the treatment of inflammatory lung diseases, including asthma.	[169]
35.	Antitumor activity	Aqueous alcoholic extract	*T. cordifolia*	Extract inhibited cell growth in a dose-dependent manner in C6 glioma cells.	Given the current research showing that TCE significantly reduced glioma cell migration and proliferation while also causing cell differentiation and programmed cell death, It's possible that this plant will show promise as a treatment for glioblastoma.	[170]
36.	Allergic rhinitis	Aqueous extract	*T. cordifolia*	Double blind placebo-controlled trial.	The age group of 20–30 and women were found to have a higher prevalence of allergic rhinitis. According to studies of the effects of TC and placebo after 8 weeks of treatment on allergic rhinitis symptoms (Table 1), patients who received TC experienced complete relief from sneezing, whereas patients in the placebo group saw no improvement at all.	[171]
37.	Diabetic neuropathy	Stem/aqueous extract	*T. cordifolia*	Aldose reductase inhibition assay in vitro and Mann-Whitney test findings from streptozotocin-induced diabetic wistar albino rats were investigated.	The herb *T. cordifolia* inhibits hyperalgesia in diabetic experimental neuropathy. The positive benefits could be attributed to its in-vitro aldose reductase inhibitory action.	[172]
38.	Hepatocellular carcinoma	Aerial parts/Ether extract	*T. cordifolia*	Male wistar rats developed hepatocellular cancer after exposure to diethyl nitrosamine.	We come to the conclusion that steryl glycosides, which are a significant component of bacteria and some plants, including cycads, can mediate nephrotoxicity through a convoluted cascade. Involves the CNS and immune system's interplay.	[30]
39.	Antimalarial activity	Stem/Ethanolic extract	*T. cordifolia*	Microorganism Models using Plasmodium berghei on white Swiss mice.	The parasite was significantly inhibited by the extract (50 %; P 0.01)	[21,173]
			40. *T. crispa*	Female ICR mice; Plasmodium berghei ANKA (PbANKA) strain that is chloroquine-sensitive; p.o.		
			41. *T. banenzigeri*			
42.	Anticancer activity	Aqueous and Ethanolic extract	*T. cordifolia*	As a model system, IMR 32 human neuroblastoma cell lines were used.	Our research suggests that this plant's active phytochemicals or crude extract could be a promising treatment option for malignant neuroblastoma cells that is based on differentiation.	[174]
		43. Ethanolic extract	*T. sinensis*	Microculture tetrazolium assay/in vitro using human melanoma cancer cell line (A 375) and skin cancer cell line (A 431).	Against the cancer cells A375 and A431, the extract demonstrated considerable cytotoxicity (IC50 values of 49.87 lg/mL and 112.54 mg/mL, respectively).	[155]
		44. Stem/aqueous	*T. crispa*	MTT assay; MCF-7 breast cancer cell lines	Significantly less cells were viable (IC50 = 42.75 mg/mL).	[158]
45.	Antibacterial activity	Stem/Aqueous and Ethanolic Extract	*T. cordifolia, T. crispa, T. sinensis*	*P. vulgaris, E. coli, S. typhi, S. aureus, E. faecalis,* and *S. marcesenses* were the microorganisms employed.	The findings show that ethanolic plant stem extracts have more efficacy than other extracts, such as chloroform and aqueous, which only partially prevented bacterial growth. This might be because ethanol extracts make the chemical components that give antibacterial action more soluble.	[175]

## 6 Bioactivity of major components

*T. cordifolia* and other *Tinospora* species possess a diverse range of bioactive chemicals. The many bioactive compounds, such as steroids, alkaloids, glycosides, diterpenoid lactones, sesquiterpenoids, flavonoids, furano diterpenoids, and aliphatic compounds, were investigated [48,72,82,52,54,55].

The plant *T. cordifolia* and other *Tinospora* species possess a wide range of bioactive compounds that contribute to its analgesic, antidyslipidemic, immunomodulatory, cardioprotective, antidiarrheal, antiulcer, neuroprotective, antidiabetic, anti-inflammatory, antioxidant, antimalarial, anticancer, and antibacterial properties.

Studies have indicated that Magnoflorine, Tembetarine, Isocolumbin, and Isoquinoline alkaloids obtained from plants had strong anticancer, antidiabetic, anti-inflammatory, and antibacterial activities [22,58,115]. *T. cordifolia* has been found to contain various forms of diterpenoids that exhibit cardioprotective properties. Secoisolariciresinol steroid and lignans exhibit anti-inflammatory and antioxidative characteristics [25].

The flavonoids apigenin and diosmetin, which have been extracted from *T. crispa*, exhibit cardioprotective, anti-inflammatory, and antioxidant properties [44,52,99]. Diosgenin, which has been found in *T. cordifolia, T. sinensis, and T. crispa*, exhibits anti-inflammatory activities [[154], [155], [156]].

## 7 Safety and toxicity profile of *T. cordifolia*

Acute poisoning tests on *T*. *cordifolia* did not show any harmful effects or deaths, even when the plant was given at a very high dose of 9 g/kg body weight [101]. According to the acute toxicity tests, giving mice a 3 g/kg dose of *T. cordifolia* aqueous extract had no negative effects [171]. Studies on humans have shown that *T. cordifolia* does not have any harmful effects, even when given at very high amounts (900 mg/day) to HIV patients and people with allergic rhinitis. Clinical studies on *T. cordifolia* showed that a dose of 500 mg/day for 21 days was safe for people and did not seem to have any negative effects on their kidneys, heart, intestines, or nervous system [25]. In a study, scientists administered a dosage of 3 g/kg of *T. cordifolia* to rats and saw no adverse effects and did not result in any fatalities among the subjects [158]. Administering a dosage of 0.1 g/kg of *T. cordifolia* to rats for 12 weeks does not adversely affect their liver or renal function. Researchers gave the drug to rats, and it increased the number of white blood cells and neutrophils. However, this did not happen in healthy people [24,159]. During long-term monitoring studies, finding and displaying signs and symptoms can give researchers important information about the right way for drugs to work and how much to give during the experiment [176]. Adding *Curcuma longa* and *T. cordifolia* to normal anti-tuberculosis treatment for all TB patients was said to greatly reduce the number and severity of liver damage events [177]. In the first part of the study, giving *T. cordifolia* to healthy volunteers was found to be safe and well-accepted [[178], [179], [180]]. Several studies have been done on the phytochemical makeup and biological qualities of different medicinal plants. According to what they found, *T. cordifolia* does not have any significant side effects, acute or chronic toxins, or LD50 values. For therapeutic reasons, it is suggested that a half-dose of this drug, or 500 mg per kg, be given. Even so, the *Tinospora* species has not been the subject of many thorough toxicological tests and safety studies. Most of the time, low to moderate doses are appropriate to use.

## 8 Marketed formulation of *T. cordifolia*

There are numerous market formulations based on *Tinospora cordifolia* that each play a unique role in sustaining health and encouraging a disease-free lifestyle. Giloy products come in a variety of formulations, including powder, syrup, juice, pills, and many others. These products appear to treat a variety of ailments and to increase immunity [101,139]. Several of these items are listed in Table 4.

**Table 4:** Marketed Formulation *Tinospora cordifolia* along with their application.

S.No	Product Name	Brand Name	Application of Product
1.	Giloy juice	Kapiva	Effective for gout, jaundice, anaemia, and fever patients. By emptying contaminants, it functions as a detoxifier, while also enhancing skin health and lowering respiratory diseases.
2.	Giloy capsules	Zandu	Improves the digestive system, regulates blood sugar levels, and keeps the liver healthy.
3.	Giloy ghanvati	Dabur	Promotes digestion, contributes to the formation of immunity, and prevents numerous illnesses
4.	Guduchi ghrita	Guduchi	Teats skin conditions and gout
5.	Giloy ghan vati	Patanjali	Aids in the treatment of gastroenteritis and offers protection from infectious disorders, persistent fever, cough, and cold
6.	Brave heart capsule	Brave Heart	Reduces blood pressure, lipid levels, particularly cholesterol and LDL cholesterol, and regulates cardiac function.
7.	Immuniveda Chyawanprash	Saffola	Cholesterol improves bioavailability, boosts immunity and respiratory health, and gives people more strength, energy, and endurance.
8.	Cirrholiv-ds syrup	Paul Medicos	Used as an immune modulator and hepatoprotectant. Treats illnesses associated to the liver
9.	Guduchi sattva	DAV Pharmacy	Effective in liver conditions, Diabetes, cough, and fever
10.	Guduchi churna	Baidyanath	Treats malaria, swine fever, and dengue, a hypoglycemic agent that aids in the treatment of diabetes, possesses anti-aging qualities that aid in enhancing skin health.
11.	Madhumehari	Baidyanath	Decreases blood and urine sugar levels, aids in energy recovery
12.	Panchanim badi churna	Prakruti Remedies	Ascites, arthritis, diabetes, eczema, dermatitis, and other skin conditions.

## 9 Conclusion and future prospective

*Tinospora cordifolia* and other species of the *Tinospora* genus are recognized as medicinal plants with recognized ethnopharmacological and therapeutic properties. It is widely observed in many clinical contexts within the Indian Systems of Medicine. T. cordifolia is a versatile medicinal plant with various types of bioactive compounds, including glycosides, alkaloids, sesquiterpenoids, aliphatic compounds, steroids, etc. The plant's anticancer, antidiabetic, analgesic, immunomodulatory, antioxidant, antiulcer, antibacterial, antipyretic, and nephroprotective effects are attributed to the presence of several bioactive compounds.

This review demonstrates that the primary areas of interest in phytochemistry, pharmacological research, and traditional clinical application on *Tinospora cordifolia* and other *Tinospora* species, including *T. crispa* (L.), *T. sinensis* (Lour.), and *T. baenzigeri*. The chemical and biological activities of other *Tinospora* species, namely *T. glabra, T. formanii, T. smilacina, T. maqsoodiana, and T. subcordata* remain unexplored or insufficiently documented. Additional research is required to elucidate the chemical diversity and fully exploit the biological importance of the chemicals and extracts derived from *Tinospora* species. However, in this regard, it is necessary to undertake additional scientific research to investigate the chemical composition and pharmacological properties of other *Tinospora* species in preventing and treating disease. This review will facilitate the future development of novel therapeutic products and help the advancement of knowledge in this particular field.

## Funding

This work did not receive any specific grant from funding agencies in the public, commercial, or not-for-profit sectors.

## Data availability statement

All data required to support this study is already mentioned in the manuscript.

## CRediT authorship contribution statement

**Anu Chaudhary:** Writing – review & editing. **Rina Das:** Data curation. **Kiran Mehta:** Formal analysis. **Dinesh Kumar Mehta:** Formal analysis, Data curation.

## Declaration of competing interest

The authors declare that they have no known competing financial interests or personal relationships that could have appeared to influence the work reported in this paper.
